# Review of the Traditional Uses, Phytochemistry, and Pharmacological Activities of *Rhoicissus* Species (Vitaceae)

**DOI:** 10.3390/molecules26082306

**Published:** 2021-04-16

**Authors:** Nondumiso P. Dube, Xavier Siwe-Noundou, Rui W. M. Krause, Douglas Kemboi, Vuyelwa Jacqueline Tembu, Amanda-Lee Manicum

**Affiliations:** 1 Department of Chemistry, Tshwane University of Technology, 175 Nelson Mandela Drive, Private Bag X680, Pretoria 0001, South Africa; DlaminiNP2@tut.ac.za (N.P.D.); kemboidouglas01@gmail.com (D.K.); 2 Department of Chemistry, Rhodes University, P.O. Box 94, Grahamstown 6140, South Africa; r.krause@ru.ac.za

**Keywords:** botany, *cuneifolia*, pharmacology, phytochemistry, *Rhoicissus*, Vitaceae

## Abstract

Species within the genus *Rhoicissus* (Vitaceae) are commonly used in South African traditional medicine. The current review discusses the occurrence, distribution, traditional uses, phytochemistry, and pharmacological properties of *Rhoicissus* species covering the period 1981–2020. The data reported were systematically collected, read, and analysed from scientific electronic databases including Scopus, Scifinder, Pubmed, and Google Scholar. Reported evidence indicates that species in this genus are used for the treatment of gastrointestinal complaints, sexually transmitted infections (STIs), and infertility, as well as to tone the uterus during pregnancy and to facilitate delivery. Pharmacological studies have further shown that members of the *Rhoicissus* genus display antidiabetic, uterotonic, ascaricidal, hepatoprotective, antioxidant, antimicrobial, anticancer, and anti-inflammatory properties. They are linked to the presence of bioactive compounds isolated from the genus. Hence, *Rhoicissus* species can potentially be an alternative therapeutic strategy to treat diseases and develop safer and more potent drugs to combat diseases. Plant species of this genus have valuable medicinal benefits due to their significant pharmacological potential. However, scientific investigation and information of the therapeutic potential of *Rhoicissus* remain limited as most of the species in the genus have not been fully exploited. Therefore, there is a need for further investigations to exploit the therapeutic potential of the genus *Rhoicissus*. Future studies should evaluate the phytochemical, pharmacological, and toxicological activities, as well as the mode of action, of *Rhoicissus* crude extracts and secondary compounds isolated from the species.

## 1. Introduction

For centuries, herbal medicines have been used worldwide to treat and prevent various ailments, particularly in developing countries where infectious diseases are endemic [1,2,3]. The World Health Organization estimates that approximately 80% of the world population uses traditional treatment methods for their primary healthcare system [4,5,6]. This is due to the increasing costs of conventional treatments, difficult access to modern health facilities, exhaustion of conventional therapies, lack of effective drugs for a serious illness, evolution of multidrug-resistant microorganisms, belief that natural products are better or safer, and cultural or spiritual preference [1,4,5,6,7,8]. This has drawn scientific and research interest toward naturally derived compounds. These are considered safe, effective, affordable, and biologically friendly, having fewer toxic side-effects than synthetic drugs [2,9,10,11]. Furthermore, the interest in natural products has yielded numerous impressive results, including the discovery of diversely sourced products with antimicrobial, anti-inflammatory, antihelminthic, antidiabetic, and anticancer activity, leading to pharmaceutical companies’ acceptance of natural products as a part of the modern and effective tools for new drugs and new drug leads [11,12].

Traditionally, *Rhoicissus* species are mainly used to enhance fertility and to facilitate delivery during pregnancy. Decoctions are taken orally to treat impotence and infertility. Women take them in the last trimester of pregnancy to ensure the good health of the mother and fetus by preventing long and complicated labour [13,14,15]. Species from this genus are also used for the treatment of cattle disease, gastrointestinal complaints, and cuts, swollen glands through warming of the roots in fire and pressing them against the glands, diarrhoea, broken bones, cuts, epilepsy, menorrhagia, renal complaints, sprained ankles, stomach ailments, and sores, in addition to as an antiemetic in children and as a general pain reliever [14,16,17,18,19].

The species from the genus *Rhoicissus* have also been reported to contain numerous secondary metabolites such as alkaloids, terpenoids, and flavonoids which display various biological effects such as anti-inflammatory, anticancer, and antioxidant activities [14]. Phytochemical investigations of *Rhoicissus* species have revealed the presence of bioactive compounds responsible for these activities. As a successful example, proanthocyanidin monomers and dimers, as well as gallic acid, isolated from *Rhoicissus tridentata* were found to stimulate smooth muscle contraction in isolated rat uterine tissue [15]. Lupenone isolated from *Rhoicissus* species has also been reported to possess antibiotic and antioxidant activity [15]. However, despite the medicinal application of this genus and the bioactive compounds isolated and identified from them, there exists no consolidation on previous and latest scientific information on the phytochemistry and biological activities of genus *Rhoicissus*. Hence, the current report presents a comprehensive literature review from 1981 to 2020. It includes the botany, geographical distribution, traditional uses, phytochemistry, and pharmacological properties of *Rhoicissus* species.

## 2. Literature Survey Databases

The data reported were systematically collected, read, and analysed from scientific electronic databases including Scopus, Elsevier, Scifinder, Research gate, ScienceDirect, and Google Scholar. Keywords such as Vitaceae, *Rhoicissus*, and traditional use were submitted during the search. Additional information on the botany, geographical distribution, traditional uses, phytochemistry, and pharmacological properties of *Rhoicissus* species was gathered from references in journal articles, book chapters, books, journal articles, and encyclopedias. The information thus obtained was critically analysed to obtain new insights and possible knowledge gaps for future research opportunities about *Rhoicissus* species.

## 3. Family Vitaceae

Vitaceae, also known as the grape family, are a medium-sized plant family with about 950 species belonging to 16 genera primarily distributed in the tropics, subtropics, and the north and south temperate zones [20,21,22,23,24,25]. It is reported that Vitaceae have a largely pantropical distribution in Asia, Africa, Australia, the neotropics, and the Pacific islands, with only a few genera in temperate regions [23,26,27]. The family is well known for containing one of the most economically important fruit crops, the grape (*Vitis vinifera* L.), as the source of wine, sultanas, currants, and raisins [24,26].

This known group of flowering plants usually features erect, prostrate, woody climbers, often with swollen or jointed nodes and herbaceous vines, or small succulent trees [21,24,28,29]. Vitaceae are readily distinguished from other angiosperm families by their unique seed morphology, inflorescences as cyme, corymb, or panicle, and leaf-opposed tendrils, enabling the family to be the most successful climbers in tropical and temperate forests [23,25,29,30]. The stomata apertures in the epidermis are bounded by two guard cells which primarily allow the rapid movement of carbon dioxide, water vapour, and oxygen in and out of the leaf [29]. The flowers are small, greenish, and inconspicuous, with a ring-like or lobed disc [28,31].

According to the phylogenic and morphological evidence, the new classification places the 950 species and 16 genera into five tribes: (i) tribe Ampelopsideae J. Wen & Z. L. Nie, trib. nov. (47 species in four genera: *Ampelopsis*, *Nekemias, Rhoicissus*, and *Clematicissus*); (ii) tribe Cisseae Rchb. (300 species in one genus: *Cissus*); (iii) tribe Cayratiaea J. Wen & L. M. Lu, trib. nov. (370 species in seven genera: *Cayratia, Causonis*, *Afrocayratia*, *Pseudocayratia*, *Acareosperma*, *Cyphostemma*, and *Tetrastigma*); (iv) tribe Parthenocisseae J. Wen & Z. D. Chen, trib. nov (16 species in two genera: *Parthenocissus* and *Yua*); (v) tribe Viteae Dumort (190 species in two genera: *Ampelocissus* and *Vitis*). The largest genera in this family are *Cissus* L. and *Cyphostemma* (Planch) [23,25,26,30]. A taxonomy of the Vitaceae family is summarised in Table 1.

## 4. Genus Rhoicissus

### 4.1. Occurance, Distribution, and Botanical Description

The genus *Rhoicissus* Planch. is one of the smallest genera in the family [26]. Approximately 12 species represent it, namely, *Rhoicissus tridentata*, *R. digitata*, *R. rhomboidea*, *R. tomentosa*, *R. revoilli*, *R. sessilifolia*, *R. microphylla*, *R. kougabergensis*, *R. laetans*, *R. capensis*, *R. erythrodes*, and *R. sekhukhuniensis*, which are endemic to tropical and southern Africa [26,27,32]. *Rhoicissus* species are distributed in the Afrotropical zone of Africa, to the south of the Sahara Desert, in the southern and eastern parts of the Arabian Peninsula, Madagascar, southwestern Pakistan, and the Islands of the Western Indian Ocean [26,30].

*Rhoicissus* species are described as climbing shrubs or woody vines, with tendrils opposite the leaves and more or less swollen nodes. The leaves are different, simple, or palmately compound. They are often rusty, with inflorescences borne opposite the leaves with 5–7 merous flowers that are small, greenish, and inconspicuous, with a ring-like or lobed disc. The stamens are equal in number to the petals and opposite to them [31,32]. The flower buds are globose in outline, while the floral disc is annular, entire, and thick [33]. Furthermore, they usually have 5–6 fleshy petals per flower. The genus can be distinguished from other genera of the tribe by its distinctly rugose seeds with linear chalaza and divergent ventral in folds [25]. The summary of phytochemistry, ethnomedicinal and biological studies reported for *Rhoicissus* species is illustrated in Table 2. 

### 4.2. Rhoicissus digitata

**Figure 1:**
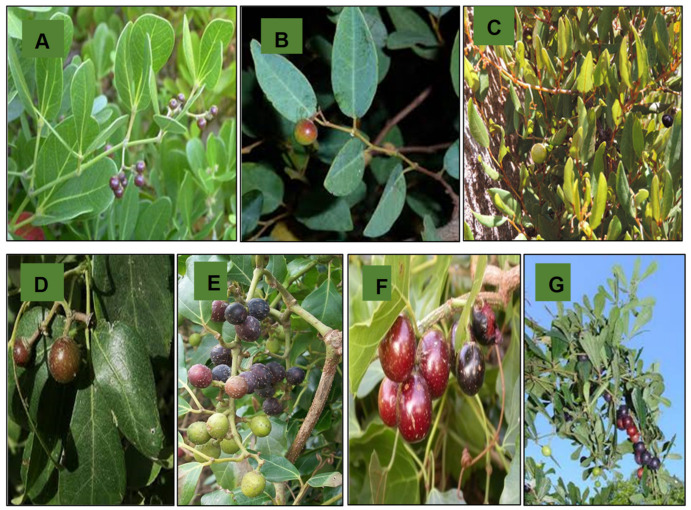
*Rhoicissus* species: (**A**) *R. digitata*; (**B**) *R. laetans*; (**C**) *R. microphylla*; (**D**) *R. revoilii*; (**E**) *R. rhomboidea*; (**F**) *R. tomentosa*; (**G**) *R. tridentata*.

*Rhoicissus digitata* (L.f.) Gilg & Brandt can grow and reach 15 m in length, is usually found in riparian fringing vegetation, and is reportedly from Natal, Transvaal (South Africa) and Mozambique [34]. This species is a vigorous, evergreen vine, climbing by leaf-opposed tendrils. Leaves are compound, usually trifoliate, although palmate compound leaves with four or five leaflets are not uncommon. Tendrils and axillary buds are present at every node and unbranched. Inflorescences are leaf-opposed and cymose, with sparsely compound dichasia on which a small number of flowers are produced. Fruits form in small clusters; they are green when unripe and turn dark purple when ripe, and they ripen from September through to December (Figure 1A) [34,35]. It is well known as baboon grape and dune grape (in English), isinwazi (in isiZulu), and vyfvingerdruif (in Afrikaans), and they are used for medicinal purposes [35].

### 4.3. Rhoicissus laetans

*Rhoicissus laetans* (Turcz.) Gilg & Brandt is a shrub up to 1.5 m high, sometimes scrambling with simple, petiolate leaves and absent tendrils [33]. Its berries are 12 mm in diameter. Classification of this species in the genus *Rhoicissus* is supported by the shape of its flower buds, structure of the floral disc, and inflorescence morphology [33]. *R. laetans* is distinguished from all the other members of the genus by its shrubby habit and simple, glabrous, glaucous green leaves (Figure 1B) [33]. *R. laetans* is endemic to the northeastern Transvaal escarpment, where it occurs in a small area. The species is found in mountain grassland with stunted shrub vegetation or steep, densely wooded kloof slopes with mixed bushveld vegetation. Occasionally, it occurs in riverine forest. The species grows on soils derived from quartzite and sandstone, and it flowers from at least November till February [33].

### 4.4. Rhoicissus microphylla

*Rhoicissus microphylla* (Turcz.) Gilg & Brandt is a small shrub which has ovate leaves with the lamina’s undersurface covered with reddish-brown hairs (Figure 1C). It occurs in the Eastern Cape, South Africa [33].

### 4.5. Rhoicissus revoilii

*Rhoicissus revoilii* Planch., commonly known as bushveld grape or warty grape, is a woody climber with tendrils about 1–10 m in height [18]. It is a shrub, robust woody climber, or a creeper in mid- to high-altitude forests, forest margins, bushveld, riverine shrubs, or woodlands [36]. This species has glossy dark green trifoliate leaves with entire margins, looping lateral veins, and domatia on lateral vein axils of the abaxial surface (Figure 1D). The leaflets are elliptic, and the laterals of this species are asymmetrical, glabrous, or hairy [36]. Their fruits are reddish to black, two-lobed, round in shape and edible [31]. It is distributed in eastern Africa from Ethiopia and Sudan to the Democratic Republic of Congo, Zambia, Zimbabwe, Mozambique, and eastern South Africa (Transvaal, Natal, Swaziland), as well as in Ghana, Comoro Islands, Saudi Arabia, and Yemen [37].

### 4.6. Rhoicissus rhomboidea

*Rhoicissus rhomboidea* (E. Mey. ex Harv.) Planch. is a canopy climber that can grow and reach 20 m [36]. It is usually referred to as bastard forest grape or rope wood grape and is distributed in Eastern South Africa (Transvaal, Natal, Swaziland, Cape Province), Mozambique [37]. This species is found in forests, forest margins, and thickets. The leaves are always made up of rhomboid leaflets (asymmetrical rhomboid for lateral leaflets) and almost always with six dentitions along the margin. It has a globose bud shape with thick spreading petals. The leaflets are shortly stalked, 1–4 mm long. It has trifoliate leaves and russet hairs on both surfaces of the leaflets. The leaves are leathery, glossy, and dark green on the adaxial surface and without hair when fully grown (Figure 1E) [36].

### 4.7. Rhoicissus tomentosa

*Rhoicissus tomentosa* (Lam.) Wild & Drummond is commonly known as an evergreen grape in English, *idiliya* in Xhosa, and i*siNwaz*i in Zulu, and it is distributed in southern Africa (Transvaal, Natal, Swaziland, Cape Province) [37]. *R. tomentosa* can either be a scrambling shrub or a canopy climber that can reach up to 20 m in height. It is found on the fringes of forests or gaps in closed forests and on riverine bushes [36]. The leaves of *R. tomentosa* are broadly transversely elliptic to reniform [33]. The leaves have lobes along the margin, simple in form, and three-nerved at the base. They are dark green on the adaxial surface and covered with rusty velvet hairs on the abaxial surface (Figure 1F). Rusty velvety hairs are also found covering young stems and tendrils but are later lost on stems as they mature and are replaced by raised dots known as lenticels [36]. Their flowers in dense axillary heads are small and yellowish-green. The fruit is edible, globose, about 20 mm in diameter, and red to purplish-black [31].

### 4.8. Rhoicissus tridentata

*Rhoicissus tridentata* is a polymorphic species with numerous nomenclature revisions over the last two centuries [38]. Originally, this species was divided and classified as several species in the genus *Rhus*. It was later reclassified in the genus *Cissus*. Later, Wild and Drummond in 1963 combined three different species, *R. cuneifolia*, *R. erythrodes*, and *R. cirrhiflora* and classified them as *R. tridentata* [20]. Urton then combined five different species in 1986, *R. cuneifolia, R. erythrodes, R. cirrhiflora, R. pauciflora*, and *R. dimidiata*, and classified them as *R. tridentata* but divided the species into two subspecies, being subspecies *tridentata* and subspecies *cuneifolia* [20,38]. *Cissus dimidiate* was noted as being a mere form of *Rhoicissus sericea*. They considered *R. sericea* to be conspecific with the plant named *Cissus dimidiata* by Ecklon and Zeyher, 1835 and correctly applied the name *Rhoicissus dimidiata* to them [20].

The subspecies are divided according to the number of indentations on the leaflet margins [38]. If the leaflets have no dentations or crenations on the leaflet margins or if the number of dentations is four or less, they are classified as subspecies *tridentata*. If the leaflet has more than four dentations, then it is classified as *cuneifolia*. The subspecies *cuneifolia* is more prevalent, having a wider distribution, extending from the Eastern Cape to the northern portion of South Africa. In contrast, subspecies *tridentata* occurs from Riversdale district eastward to Port St Johns and then extends inland in the Southeastern Cape to the Karoo of South Africa [20].

*R. tridentata* (L.f) Wild & Drumm. subsp. *cuneifolia* (Eckl. & Zehr.), N.R. Urton is a deciduous shrubby creeper in the Vitaceae family. It is commonly known as wild grape (English), isinwazi (Zulu), and umnxeba (isiXhosa) [39]. This subspecies occurs in various habitats but is primarily found in forests along forest margins or grows as an erect shrub of up to 2 m or more in open grassy woodlands [20]. *R. tridentata* has tendrils and can grow up to 3 m high, spreading up to 1.5 m. The leaves are trifoliate with wedge-shaped leaflets, each having a serrated margin (Figure 1G). The plant bears small inconspicuous yellowish-green flowers followed by small brownish-red berries [39]. Lignotubers ranging in size from 5 to 30 cm in diameter are attached to the roots [20].

## 5. Ethnomedicinal Uses of *Rhoicissus* Species

Species of the *Rhoicissus* genus are widely used in traditional medicines in African medicinal systems to treat various diseased conditions. According to several reports, they are common medicinal herbs used by the Zulus and Xhosas of South Africa. They are used to treat cattle diseases, high blood pressure and acute headaches, as well as for blood purification and intestinal cleansing, increasing fertility, relieving menstruation pain, managing helminthiasis and venereal diseases, treating bloody constipation, increasing milk production in lactating mothers, wound and ringworm healing, anaesthetic properties, and facilitating delivery during pregnancy, among others [14,40,41,42,43], as summarised in Table 3. However, no particular class of compound has been identified or linked to a particular medicinal application. Furthermore, even though most of the *Rhoicissus* species are used in African traditional medicine, their prescription, mechanisms and mode of actions, associated side-effects, and/or proven efficacy are not clear. A more detailed study is needed to establish their mode of action, side-effects, and safety.

## 6. Biological Studies of *Rhoicissus* Species

### 6.1. Anti-Inflammatory Activity

The research reported on the anti-inflammatory potential of methanolic extracts of *R. digitata* (leaf), *R. romboidea* (root), *R. tomentosa* (leaf/stem), and *R. tridentata* (root) indicated significant inhibition of cyclooxygenase (COX-1) [59,60]. COX-1 is a prostaglandin-producing enzyme that promotes inflammation, pain, and fever, activates platelets, and protects the stomach and intestinal lining. An ethanol indomethacin standard solution (20 µM in the assay) was assayed together with the samples (13.3 mg for each sample residue) to verify the sensitivity of the assay [59]. The extracts of *R. digitata* and *R. rhomboidea* exhibited the highest inhibition of prostaglandin synthesis with 53% and 56% inhibition, respectively, compared to 89% inhibition by the indomethacin standard, suggesting their potential to be used as anti-inflammatory agents [59,60]. It was reported that none of the aqueous extracts showed any significant anti-inflammatory activity [59]. Furthermore, *R. tridentata* was investigated with the anti-inflammatory enzyme 15-lipoxygenase (15-LOX). The enzyme (15-LOX) was made up to a final concentration of 200 units/mL in 2 M borate buffer (pH 9), and a volume of 12.5 μL of each plant sample and control was added to 487.5 μL of 15-LOX [52]. *R. tridentata* showed a half maximal inhibitory concentration (IC_50_) of 87.39 µg/mL against the 15-LOX enzyme [52]. The results suggested that members of the genus *Rhoicissus* have the potential to be used as anti-inflammatory agents.

### 6.2. Antimicrobial Activity

The antimicrobial activities against Gram-positive and Gram-negative microorganisms of the methanolic extracts of *R. digitata*, *R. rhomboidea*, *R. tomentosa*, and *R. tridentata* were investigated [59]. Standard antibiotics, penicillin G (10 IU·disc^−1^), tetracycline (30 µg·disc^−1^), and chloramphenicol (30 µg·disc^−1^), were used to eliminate variations between plates [59]. It was found that all extracts showed some degree of antimicrobial activity, with *R. rhomboidea* (root) demonstrating the highest inhibitory activity against different microorganisms. The crude methanolic extracts of *R. tridentata* and *R. digitata* mainly inhibited Gram-positive and Gram-negative microorganisms. Most of the extracts showed insufficient inhibitory activity towards *Salmonella* sp. including *S. Typhimurium* and *Shigella* sp. (*S. boydii* and *S. flexneri*). None of the extracts inhibited *Escherichia coli* or showed activity against *S. flexneri*. *R. digitata* (leaf) extract indicated a >10.00 mm inhibition zone diameter against *S. boydii* compared to the standard chloramphenicol that showed a 7.00 mm inhibition zone diameter. Tetracycline showed no activity against *S. boydii* [59] as a standard. *R. rhomboidei* stem extract and the standards chloramphenicol and tetracycline all indicated a >10.00 mm inhibition zone diameter against *Salmonella sp*. [59]. It was also reported that the plant extracts inhibited the Gram-positive microorganisms more than the Gram-negative ones [59].

The antifungal activity of *Rhoicissus* species was tested using *Candida albicans* and *Saccharomyces cerevisiae* [59]. Methanolic extracts of *R. digitata* (leaf) and *R. romboidea* (root) exhibited the highest antifungal activity against *C. albicans* with an inhibition zone diameter of >10.00 mm. A methanol extract of *R. tridentata* exhibited antifungal activity against *C. albicans* with an inhibition zone diameter of 7.00 mm [59].

A study conducted to screen 14 medicinal plants used by the Venda community for infectious diseases supported the antibacterial activities of the methanol extracts of the roots and tubers of *R. tridentata* [61]. Experiments were conducted using 10 µL of a 50 mg/mL gentamycin solution as a positive control and 15 µL (6%) of dimethyl sulfoxide (DMSO) as a negative control [61]. The extracts of *R. tridentata* showed activities against all the organisms tested with minimum inhibitory concentrations (MICs) varying from 0.75 mg/mL against *Bacillus cereus* to 6.00 mg/mL against *Bacillus subtilis*, *Enterobacter cloacae*, and *Pseudomonas aeruginosa*. The minimum inhibitory concentration of gentamycin was 0.008 mg/mL against most of the organisms tested and 0.017 mg/mL for *P. aeruginosa* and *Serratia marcescens* [61]. Fruit extracts were not as active as the underground parts and exhibited MICs of more than 12.00 mg/mL [61].

Antimicrobial properties of the methanol root and leaf extracts of *R. revoilli* were also studied. The extracts were active against three microorganisms: Gram-positive *Streptococcus pyogenes*, Gram-negative *Salmonella typhi*, and the fungal pathogen *Aspergillus niger* [47]. First, 3 g of the filtered and dried plant extract was constituted with 10 mL of 100% cyclohexane to prepare a stock solution. The control had cyclohexane alone without any extract to cancel the effect of the solvent on the test organisms [47]. The growth inhibition diameter was 4.68 mm for *S. pyogenes*, 5.08 mm for *S. typhi*, and 4.27 mm for *A. niger*. The root extract showed more significant microbial growth inhibition in comparison to leaf extracts [47]. *R. revoilli* also displayed inhibitory activity against *Staphylococcus aureus* at high dilution levels with a 9.00 mm inhibition zone diameter and showed minimal inhibitory activity against *Escherichia coli* (8.00 mm inhibition zone diameter) [62]. The standard, Dettol, showed higher inhibitory activity against *E. coli* (20.00 mm inhibition zone diameter) but only at low dilution levels, and inhibitory activity against *S. aureus* was lower than in the case of *E. coli* with a 12.00 mm inhibition zone diameter [62]. The ethanol extract of *R. revoilii* rhizomes displayed inhibition zone diameters of 12.82 mm against *E. coli* and 17.50 mm against *C. albicans* [63]. Standard concentrations of 0.12 mg/mL nystatin and 0.3 mg/mL chloramphenicol were used as positive controls. About 500 mg of the ethanol extract was triturated with 1 mL of DMSO and then made up to 5 mL in distilled water to give a test solution of 100 µg/µL concentration for each fraction [63]. These results support the traditional antimicrobial use of *R. revoilli*.

Different medicinal plants were tested for antifungal activities against five *Fusarium* species using the whole-plate diffusion method [64]. Sterile dimethyl sulfoxide (DMSO) was used as a negative control, and nystatin was used as a positive control [64]. The methanol extract (roots) of *R. tridentata* was active against *Fusarium graminearum* with an inhibition zone diameter of 15.00 mm compared to an inhibition zone diameter of 20.00 mm for nystatin [64]. The acetone extract (tubers) showed high activities with MIC values ranging between 0.95 and 3.75 mg/mL against the five *Fusarium* species tested [64].

Methanol/chloroform (50/50, *v*/*v*) and ethyl acetate (100%) extracts of the rhizomes of *R. tomentosa* were tested against 14 bacterial strains using the disc diffusion and microdilution assay methods [46]. Bacteria most susceptible to rhizome extracts were *Staphylococcus aureus* (MIC of 0.06 mg/mL) and *Bacillus subtilis* (MIC of 0.13 mg/mL) [46]. The results showed that the rhizome extracts of *R. tomentosa* have good antibacterial activity against Gram-positive organisms such as *S. aureus*, *B. subtilis*, *Enterococcus faecalis* (2.00 mg/mL), *Mycobacterium smegmatis* (0.06 mg/mL)*, Bacillus cereus* (0.50 mg/mL), and *Staphylococcus epidermidis* (2.00 mg/mL). In contrast, only the Gram-negative organisms *Proteus vulgaris* (8.00 mg/mL) and *Proteus mirabilis* (16.00 mg/mL) displayed sensitivity to the extracts [46].

Extracts of selected plant species used to treat sexually transmitted infections (STIs) in southern Africa were investigated for antimicrobial properties and anti-human immunodeficiency virus (HIV) activity against the recombinant HIV-1 enzyme [52]. Doxorubicin at 100 µg/mL was used as the positive control. For the negative control, only the lysis buffer and reaction mixture were added [52]. *R. tridentata* demonstrated the best antimicrobial activity against *Neisseria gonorrhoeae* with the lowest MIC value of 0.40 mg/mL. *C. albicans* and *Gardnerella vaginalis* had MIC values of 0.80 mg/mL and *Oligella ureolytica* had an MIC value of 1.60 mg/mL [52]. *R. tridentata* also had the best HIV-1 RT inhibition activity of 75.50% compared to that of the positive control doxorubicin (96.50%) at 100.00 µg/mL [52]. The observed activities may lead to new multitarget drugs against STIs.

*R. tridentata* (ethanol extract) also displayed activity against *C. albicans*, *E. coli*, and *S. aureus* with an MIC of 0.80 mg/mL compared to the positive control ciprofloxacin with an MIC of 0.1 mg/mL [65].

In a related study, traditional medicinal plants used in South Africa to treat urinary tract infections caused by microorganisms were investigated [66]. Ciprofloxacin was used as the positive control at a final concentration of 0.063 mg/mL, while 100 µL of 1.0% DMSO (instead of plant extract) was used as the negative control [66]. The methanol extract of *R. tridentata* displayed the highest activity against *Serratia marcescens* with an MIC of 0.13 mg/mL [66]. The positive control ciprofloxacin presented an MIC of <0.063 mg/mL. The polar extracts of *R. tridentata* were able to reduce the initial cell attachment of *S. aureus*, *P. mirabilis*, and *Serratia marcescens* by approximately 50% [66]. The quantitative anti-quorum sensing assay indicated that the methanol extract of *R. tridentata* inhibited violacein production by *C. violaceum* by more than 50%, with an IC_50_ of 2.58 mg/mL [66]. The positive control eugenol was 1.73 mg/mL [66]. The results validate the use of *R. tridentata* to treat urinary tract infections (UTIs) and indicate that this plant may be a suitable source of antipathogenic drugs to treat UTIs [66].

### 6.3. Antiproliferative Activity

Aqueous and methanol extracts of *R. digitata*, *R. rhomboidea*, *R. tomentosa*, and *R. tridentata* were screened to determine their therapeutic potentials as anticancer agents [14]. The antiproliferative activity in vitro against HepG2 cells, a human liver cancer cell line, was determined. The aqueous root extract of *R. tridentata* subsp. *cuneifolia* displayed the highest antiproliferative activity, with a 96.27% inhibition of proliferation compared with the other crude plant extracts [14]. The methanol extract of *R. tridentata* also presented a more potent inhibition of 87.01%. The crude root extract of *R. tomentosa* exhibited 70.40% inhibition of proliferation [14]. The results from this study demonstrated that all the *Rhoicissus* species screened have potential antineoplastic activity against the HepG2 cell line, and the root extracts demonstrated stronger inhibitory activities compared with the leaf and stem extracts [14].

### 6.4. Antioxidant Activity

The antioxidant activity of *R. tridentata* was 0.06 µg/mL, lower than that of vitamin C (IC_50_ of 1.44 μg/mL) [65]. In this study, 2 mg plant samples were tested at concentrations ranging from 500–3.91 μg/mL. Ascorbic acid (vitamin C) was used as a positive control, and ethanol was used as a solvent control (blank) [65].

Methanol extracts of the roots, stems, and leaves of four *Rhoicissus* species (*R. digitate*, *R. rhomboidea*, *R. tomentosa*, and *R. tridentata*) were tested for antioxidant activity [67]. Commercial antioxidants vitamin E, butylated hydroxytoluene (BHT), and hydroxyanisole (BHA) were used as standards [67]. The extracts of *R. rhomboidea* and *R. tridentata* revealed more than 50% antioxidant activity compared with values obtained for the commercial antioxidants. The commercial antioxidants gave the following results vitamin E, 63%; butylated hydroxytoluene (BHT), 50.10%; butylated hydroxyanisole (BHA), 42.50% [67]. *R. rhomboidea* and *R. tridentata* inhibited the 1, 1′-diphenyl-2-picrylhydrazyl free radical with approximately 98% radical scavenging activity. Xanthine oxidase was inhibited by 88.20% by the root extract of *R. rhomboidei*. The stem of *R. tridentata* exhibited an inhibitory effect above 70% against xanthine oxidase. It also prevented the production of thiobarbituric acid-reactive substances and free radical-mediated DNA sugar damage (catechin showed an 85.40% inhibitory effect) [67].

The four *Rhoicissus* extracts had a strong chelating effect on Fe^2+^ ions, especially the leaves of *R. tridentata* [67]. *R. digitata* and *R. tomentosa* extracts possessed pro-oxidative properties at high concentrations (2.50 mg/100 mL) due to the presence of the plant phenolics [67]. The results indicate that the four *Rhoicissus* species have a protective action against the overproduction of free radicals. One of the mechanisms of action suggests that *R. rhomboidea* and *R. tridentata* contain compounds (polyphenols) with strong radical-scavenging and antiradical-generating effects.

### 6.5. Uterotonic Activity

Pharmacological investigation of crude aqueous extracts of *R. tridentata* roots displayed direct contractile responses in isolated rat uterus and ileum [68]. The muscarinic receptor system and the produced cyclooxygenase metabolites facilitated the contractile response to the extract [68]. The results provide evidence justifying the ethnomedical use of this plant to promote quick and uncomplicated labour. The potential exists for the plant decoctions to cause birth complications caused by increased uterine contractility [68]. It was also confirmed that the contractile activity of *R. tridentata* extracts varies seasonally to different plant parts [51]. The activity of the plant extracts from plants harvested in summer and autumn were 4–5-fold higher than extracts from plants harvested in winter or spring. The lignotubers stimulated the most significant number of contractions, followed by the stems, roots, and leaves [51].

Aqueous extracts of the roots of *R. tridentata* revealed notable in vitro activity on isolated rat uterine smooth muscle tissue [15]. Extracts with the highest activity also contained proanthocyanidin monomers, dimers, gallic acid, and 74% polymeric proanthocyanidins [15]. Glucose and a hydrogel of glucose extracted from the methanol root extract also greatly stimulated uterine muscle contraction [15]. *β*-Sitosterol and its glucoside, sitosterolin, exhibited only slight oestrogenic activity, increasing the response of the uterus by about 2% at concentrations of acetylcholine below 10^−7^ M and 10^−5^ M, respectively, and inhibiting this response at higher acetylcholine concentrations [15].

Furthermore, the chloroform and ethanol extracts of the root bark of *R. tridentata* were investigated for their in vitro activity on the contraction of corpus cavernosum smooth muscle of white New Zealand rabbits [56]. The extracts stimulated dose-dependent relaxation in the muscle at concentrations of 13.00 and 6.50 mg/mL [56]. At an extract concentration of 13.00 mg/mL, the relaxation induced was significantly higher (*p* < 0.01, 30.70 ± 3.3 chloroform extract and 43.00 ± 9.4 ethanol extract) than that seen with 3.2 × 10^−5^ mg/mL of the positive control Viagra [56]. This study indicated that *R. tridentata* is a promising candidate for the treatment of erectile dysfunction.

### 6.6. Cytotoxicity

The ethanol extract of *R. tridentata* showed moderate toxicity with a half maximal effective concentration (EC_50_) of 88.50 ± 0.09 μg/mL, whereas actinomycin D exhibited an IC_50_ value of 0.00932 μg/mL [65]. Actinomycin D ranging from 400–3.13 and 0.013 to 0.0001 μg/mL was used as a positive control for the cytotoxicity assay. The toxicity effects of the samples were determined using 50 μL of XTT reagent (1 mg/mL 2,3-bis-(2-methoxy-4-nitro-5-sulfophenyl)-2*H*-tetrazolium-5-carboxanilide (XTT) with 0.383 mg/mL *N*-methyl dibenzopyrazine methyl sulphate (PMS)) [65].

The cytotoxicity of the aqueous extracts from *R. tridentata* was studied using monkey Vero cells and human fibroblasts [50]. The threshold for zero cell deaths was 8.00 μg/mL for monkey Vero cells. At this concentration, 100% of human fibroblast cells also survived [50]. The estimated concentration in the bloodstream was 6.10 μg/mL, taking dilutions into account. It was then concluded that *R. tridentata* is not toxic at a cellular level since the estimated cell concentrations were below the thresholds for zero cell death for monkey Vero cells, which is 8.00 μg/mL [50]. The nontoxic nature of this species was also demonstrated using human hepatoma, kidney epithelial, histiocytoma, and mouse Leydig cells [15].

### 6.7. Hepatoprotective Activity

The identity of catechins and the in vitro antioxidative properties of *R. tridentata* motivated a study to investigate the in vivo hepatoprotective effects of *R. tridentata* against CCl_4_-induced acute liver injury in rats [53,55,67]. The variables investigated were the alanine aminotransferase (ALT), aspartate aminotransferase (ASP), glucose-6-phosphatase (G-6-Pase), and lipid peroxide (LPO) levels of liver homogenates. Liver microsomal fractions were investigated as malondialdehyde (MDA) levels [53]. The results displayed a decrease in the concentration of ALT, ASP, and LPO (*p* < 0.05) after the administration of the plant extract to the CCl_4_-intoxicated rats. In contrast, the G-6-Pase concentration was elevated (*p* < 0.05) in the plant extract-treated rats, and this shows that *R. tridentata* has components with hepatoprotective properties [53].

### 6.8. Ascaricidal Activity

The in vitro anthelmintic activity of the extracts of *R. tridentata* was tested, and the median effective dose (ED_50_) values of the extracts were determined using the Ascaris model [54]. The ED_50_ of *R. tridentata* was found to be 4.36 mg/mL [54]. The highest dose of 10.00 mg/mL first showed activity at 12 h, achieving a maximum response of 100% at 24 h. Doses of 4.00, 6.00, and 8.00 mg/mL first showed activity at 12 h. The maximum response was achieved at 48 h for doses of 4.00 and 6.00 mg/mL. After 36 h, the maximum response was achieved for 8.00 mg/mL. The lowest dose of 2.00 mg/mL started a response at 24 h and only achieved a 90% response at 48 h [54]. These results show that *R. tridentata* can be used for the treatment of helminth diseases in cattle.

A study to determine in vitro ascaricidal activity of ethanolic and water extract of root-tuber *R. tridentata* against adult nematodes was conducted [69]. The in vitro adult motility inhibition assay revealed that the two extracts exhibited motility inhibition, and the ethanolic extract was more potent [69]. The ascaricidal single-dose effect increased with increasing concentration of the extract. The highest concentrations of 64.00 and 128.00 mg/mL for ethanol and water extracts gave a maximum mean percentage ascaricidal activity by 48 h as 80.00 ± 10.0% and 90.00 ± 0.6%, respectively [69]. The crude extract’s potential to control gastrointestinal nematodes was indicated by the low ED_50_ of 25.00 mg/mL [69]. 

### 6.9. Antidiabetic Activity

A study was designed to evaluate the antidiabetic potential of aqueous leaf extracts of *R. tridentata* in alloxan-induced diabetic mice [19]. The aqueous leaf extracts showed antidiabetic activity. Intraperitoneally and orally administered aqueous whole-stem extracts of *R. tridentata* decreased the blood glucose levels at all four doses of 50.00, 100.00, 200.00, and 300.00 mg/kg body weight, from the first hour to the sixth hour in a dose-independent manner [19]. The intraperitoneal route of herbal extract administration was more effective than the oral route [19].

## 7. Phytochemistry of Some Species of the *Rhoicissus* Genus

The study of chemical compounds found in plants is essential for drug discovery and for developing novel therapeutic agents against significant diseases as they are biologically active. Different species of the *Rhoicissus* genus have been shown to possess bioactive compounds such as coumarins, flavonoids, phytosterols, essential oils, saponins, terpenoids, alkaloids, reducing sugars, and tannins, which are the reason for their use in traditional medicine. The phytochemical screening carried out on the rhizomes of *R. tomentosa* revealed the presence of many known groups of bioactive compounds: alkaloids, flavonoids, saponins, steroids, reducing sugars, and tannins [46].

While there have been many investigations on the pharmacological activities of this genus, little has been achieved concerning the isolation and identification of bioactive compounds. It is established that few chemical constituents have been isolated from these species within the review period, as most of the chemical constituents have been profiled from the crude extracts. This limits further studies aimed at advancing the bioactive constituents to clinical trials.

**Figure 2:**
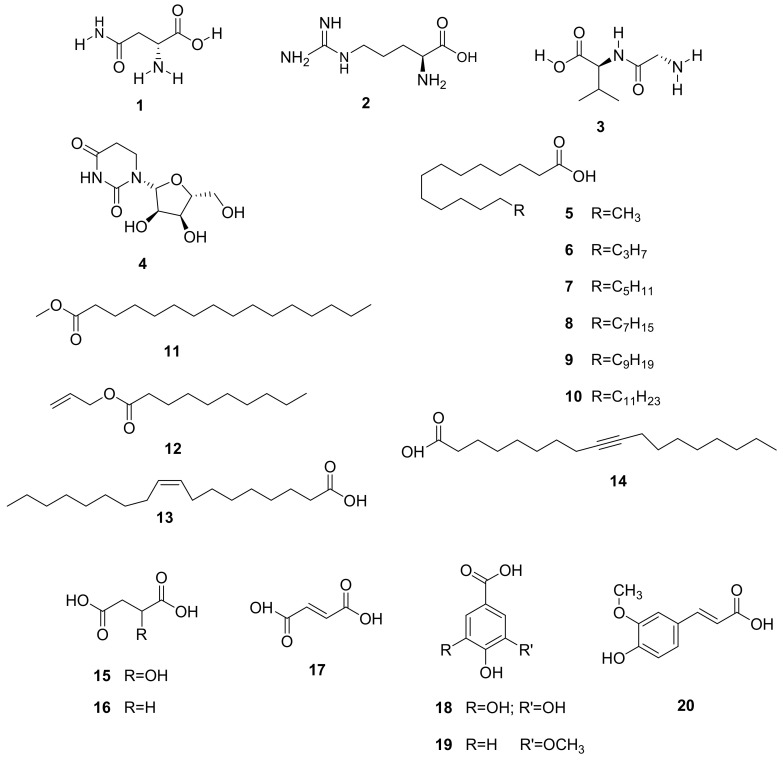
Chemical structures of acids isolated from the genus *Rhoicissus*.

**Figure 3:**
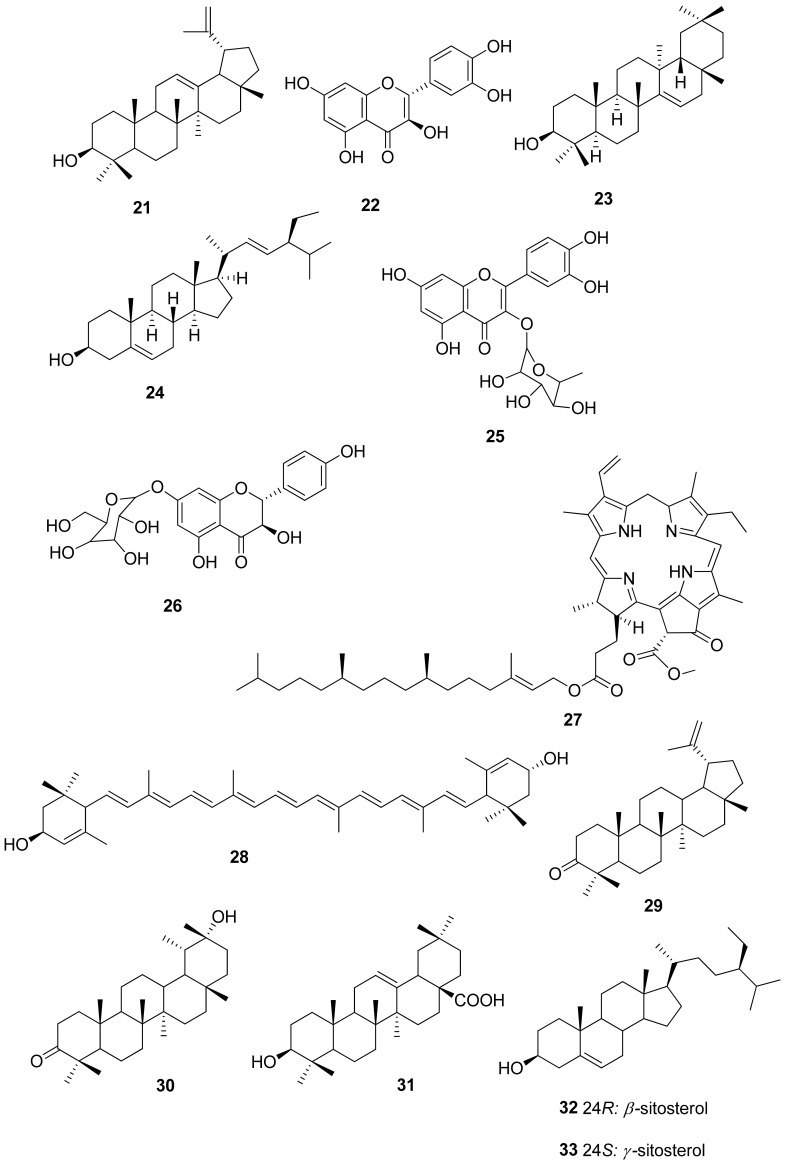
Chemical structures of triterpenoids, flavonoids, alkaloid, and carotenoid isolated from the genus *Rhoicissus*.

**Figure 4:**
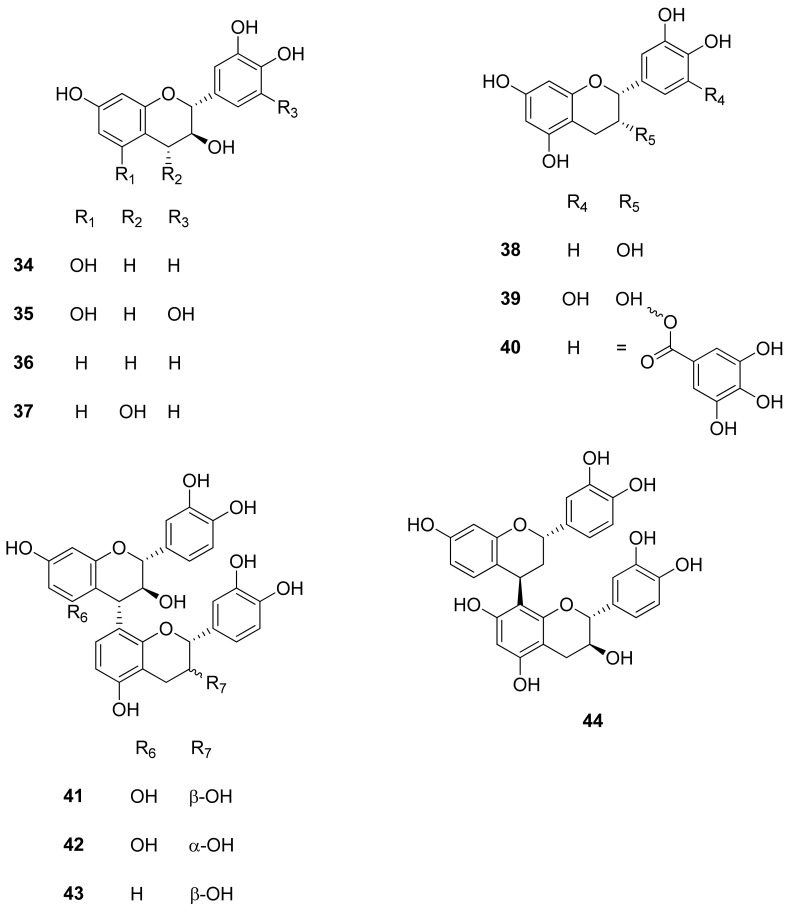
Structures of proanthocyanidin monomers and dimers from genus *Rhoicissus*.

Among the identified compounds, essential oils and other phenolic acids (**1–14**) were predominant. Other compounds included triterpenoids such as 12,13-dehydrolupeol (**21**), 3β-taraxerol (**23**), and stigmasterol (**24**), as well as flavonoids such as quercetin (**22**), quercetrin (**25**), and aromadendrin-7-*O*-β-glucopyranoside (**26**), among others, as illustrated in Table 4 and Table 5 and Figure 2, Figure 3 and Figure 4.

Organic acids (malic (**15**), succinic (**16**), and fumaric acids (**17**)) (Figure 2) were also detected in the free phenolic fraction and malonoic and propanoic acids were detected in the hydrolysable fraction of *R. tridentata* [17]. Homovanillyl alcohol was found in the free phenolic fraction, and gallic (**18**), vanillic (**19**), and ferulic acids (**20**) (Figure 2) were confirmed in the bound phenolic fraction [17]. Qualitative and quantitative phytochemical screening of the aqueous leaf extracts of *R. tridentata* also indicated the presence of phenols, alkaloids, flavonoids, tannins, and saponins [19]. *R. tridentata* is known to contain a high concentration of polyphenolic compounds [55,67]. The phytochemical screening of *R. revoilli* revealed active compounds such as flavonoids, alkaloids, saponins, steroids, and anthraquinones. Aldehydes were detected in the leaf extract [47]. However, these compounds were not structurally elucidated.

A phytochemical investigation of the roots and fruits of *R. digitata* led to the isolation of triterpenes and flavonoids [44]. From the roots, leaves, and fruits of *R. tomentosa*, terpenoids, flavonoids, an alkaloid, and a carotenoid were isolated, as illustrated in Table 5 and Figure 3 [44]. Moreover, the leaf extracts of *R. tomentosa* tested positive for coumarins, flavonoids, phytosterols, essential oils, saponins, terpenoids, and resveratrol, and the hexane and ethyl acetate/hexane (1:4) extracts of *R. tomentosa* were submitted for GC–MS analysis [83].

Previously undescribed compounds from the species were isolated from the extracts of *R. tridentata* [15]. The proanthocyanidin monomers and dimers from the methanol root extract were identified as follows using HPLC: catechin (**34**), gallocatechin (**35**), fisetinidol (**36**) mollisacacidin (**37**), epicatechin (**38**), epigallocatechin (**39**), epicatechin-3-*O*-gallate (**40**), procyanidin B3 (**41**), procyanidin B4 (**42**), fisetinidol (4α-8) (**43**), fisetinidol (4*β*-8), catechin (**44**) (Figure 4), andgallic acid (**18**) (Figure 2).

Glucose and a partially identified hydrogel of glucose were also isolated from the methanol root extract. Oleanolic acid (**31**) was isolated from a chloroform extract. The nonpolar fraction yielded two further triterpenoids, 20(29)-lupen-3-one (**29**) and 20-epi-ψ-taraxastananol (**30**), as well as *γ*-sitosterol (**33**), which were identified by gas chromatography–mass spectrometry (Figure 3). From the extract of branches, the plant growth hormone triacontanol was purified [15].

Most of the isolated compounds have documented health-promoting properties. It supports their use by traditional healers to promote good health during pregnancy. Proanthocyanidins are potent antioxidants beneficial for the heart, cardiovascular, and immune systems (Figure 4). Lupenone (**29**) has antibiotic and antioxidant activity [93]. Triacontanol has cholesterol-lowering properties, and the triterpenoids such as oleanolic acid (**31**), lupenone (**29**), and taraxastananol (**30**) are well known for their anti-inflammatory properties [15,94].

## 8. Conclusions

*R. tridentata* is used to treat various ailments including erectile dysfunction, pains, swelling, cuts, wounds, kidney and bladder complications, stomach ailments, and livestock diseases, as well as for gynaecological purposes. Compounds isolated from *R. tridentata* include proanthocyanidin monomers and dimers, phenols, alkaloids, flavonoids, tannins, saponins, organic acids, and triterpenoids. Pharmacological studies indicated that *Rhoicissus* species exhibited antitumor, antispasmodic, antipruritic, anaesthetic, neuroprotective, analgesic, antipyretic, antidepressant, antiepileptic, anthelmintic, antiasthmatic, antidiabetic, uterotonic, ascaricidal, hepatoprotective, antibacterial, antidiabetic, antioxidant, antimicrobial, antifungal, anticancer, and anti-inflammatory properties. These results are encouraging and indicate that *Rhoicissus* is an essential source of many pharmacologically and medicinally important compounds. Hence, this plant has the potential as an alternative therapeutic strategy to treat diseases and can be used as part of a template for developing safer and powerful drugs to combat diseases. The observed biological activities of the members of *Rhoicissus* support the medicinal use of these plants by traditional healers. However, no specific compound class has been identified or linked to a particular ethnomedicinal application of these species. Their prescription, mechanisms, associated side-effects, and/or proven efficacy are also not clear. Thus, there is a need for more detailed studies to establish the missing information.

While many investigations into the pharmacological activities of the genus *Rhoicissus* have been done, little has been achieved concerning the isolation and identification of bioactive compounds. Only a few chemical constituents have been isolated from these species within the review period. This limits further studies aimed at advancing the bioactive constituents to clinical trials. Studies focusing on the biological activities of *Rhoicissus* have been conducted in vitro, and little data are available on the biological activities of compounds isolated from the species. There is a need for further studies focusing on the phytochemistry, pharmacological, and toxicological properties, as well as in vivo studies involving the crude extracts and chemical compounds isolated from the species. New techniques such as high-throughput screening, molecular docking studies, and metabolomics should be advanced, especially for complex plant matrices.

## Figures and Tables

**Table 1:** Taxonomy of the Vitaceae family.

Taxonomic Hierarchy	Classification	Reference
Kingdom	Plantae—plants	[23,25,26,30]
Subkingdom	Tracheobionta—vascular plants	[23,25,26,30]
Super division	Spermatophyta—seed plants	[23,25,26,30]
Division	Magnoliophyta—flowering plants	[23,25,26,30]
Class	Magnoliopsida—dicotyledons	[23,25,26,30]
Subclass	Rosidae	[23,25,26,30]
Order	Rhamnales	[23,25,26,30]
Family	Vitaceae—grape family	[23,25,26,30]

**Table 2:** Summary of phytochemistry, ethnomedicinal, and biological studies reported for *Rhoicissus* species.

Plant Name	Botanical Description	Traditional Uses	Biological Activity	Phytochemistry
*R. capensis*	Not reported	Not reported	Not evaluated	Not evaluated
*R. digitata*	Reported	Reported	Evaluated	Evaluated
*R. erythrodes*	Not reported	Not reported	Not evaluated	Not evaluated
*R. kougabergensis*	Not reported	Not reported	Not evaluated	Not evaluated
*R. laetans*	Reported	Not reported	Not evaluated	Not evaluated
*R. microphylla*	Reported	Not reported	Not evaluated	Not evaluated
*R. revoilli*	Reported	Reported	Evaluated	Evaluated
*R. rhomboidea*	Reported	Reported	Evaluated	Not evaluated
*R. sekhukhuniensis*	Not reported	Not reported	Not evaluated	Not evaluated
*R. sessilifolia*	Not reported	Not reported	Not evaluated	Not evaluated
*R. tomentosa*	Reported	Reported	Evaluated	Evaluated
*R. tridentata*	Reported	Reported	Evaluated	Evaluated

**Table 3:** Traditional medicinal uses reported for *Rhoicissus* species.

Plant Name	Ethnic Name (Z—Zulu; X—Xhosa)	Part	Traditional Use	Reference
*R. digitata*	Isinwazi (Z); Uchititibhunga (X,Z); umNangwazi (Z); umPhambane (Z); iTangalehlathi (Z); umThwazi (Z)	Leaf	During pregnancy, to facilitate delivery, isihlambezo and inembe, which is taken as an abortifacient.	[14]
		Tubers	It is used to treat cattle diseases.	[40]
			An infusion of the tuber is taken for high blood pressure and acute headaches; It is used to treat goats and sheep with paratyphoid.	[41]
		Bulbs	Enema for blood purification and intestinal cleansing; Used to treat gastrointestinal complaints; Used as an amulet that destroys gossip; The plant is also used to increase fertility; Used to treat painful menstruation; Used as a general pain reliever.	[42,43]
		Roots	Used in the preparation of stomach medicine.	[16,44]
*R. rhomboidea*	Isinwazi (Z)	Roots	Used during pregnancy to facilitate delivery.	[14]
*R. tomentosa*	Isinwazi Idiliya (X); Impindabamshaye (X); UmPhambane (Z)	Leaf/ stem	Anthelminthic for calves, during pregnancy to ensure a safe delivery, and dysmenorrhoea.	[14]
		Bark	Used for heartwater in livestock by crushing and boiling in water and used with *Kedrostis africana* to treat 3 day stiff sickness.	[45]
		Roots	Crushed and boiled in milk for young calves to treat or manage helminthiasis.	[45]
			The boiled roots fusion are used to enhance fertility.	[42,44,46]
			To treat goats and sheep with paratyphoid.	[41]
*R. revoilii*	Isinwazi (Z)	Roots	A root decoction is taken as a remedy for venereal diseases and bloody constipation.	[18]
			Decoctions are given to breastfeeding mothers and cows to increase milk production.	[47]
		Stem	Sap from the stem is applied to cuts, burns, and sores.	[18]
		Leaves (crushed)	They are externally rubbed onto infected skin to hasten wound and ringworm healing. Decoctions are orally taken to treat intestinal worms, including hookworms. Decoctions are also externally applied to boils to ensure faster healing.	[47]
			Used as an antiseptic.	[18]
			Fresh leaf and stem squeezed together with water and given orally and also nasally for livestock to treat leech infection.	[48]
*R. tridentata*	Isinwazi (Z)	Roots	Used as herbal oxytocics.	[15]
			Used with *Clivia miniate*, *Agapanthus africanus*, *Pentanisia prunelloides*, and *Gunnera perpensa* to prepare isihlambezo (that which cleans) decoctions used by women in the last trimester of pregnancy.	[13,15,49,50,51]
			Ease of ingestion by use of juice from the roots extracted through chewing, care of abdominal pain during menstruation, treatment of swollen glands through warming of the roots in fire and pressing them against the glands, antiemetics in children, broken bones, cuts, epilepsy, menorrhagia, eye infections, sexually transmitted infections (STIs), sprained ankles, stomach ailments, and sores.	[14,17,19,52]
			Used in the protection of liver damage, also known as hepatoprotective effects.	[53]
			Its sap is reported to have healing and anaesthetic properties, and tuberous roots are boiled and fed to young animals, especially those that have lost their mothers.	[19]
			Used for the treatment of helminth diseases in cattle and the tick-borne cattle disease, babesiosis.	[54,55]
			Used to treat erectile dysfunction.	[56]
			Heartburn, peptic ulcers, diarrhoea, renal disorders, and infertility in women.	[57]
			Heartwater, redwater, internal parasites, general ailments, and abortion.	[58]

**Table 4:** Compounds identified in *R. tomentosa* rhizomes and their biological activities.

Compound Number	Name of Compound	Biological Activity	Reference
1	d-Asparagine	Immuno-stimulant, antibacterial, anti-infective, and analgestic activities.	[70]
2	l-Arginine	Anti-inflammatory, immunostimulant, and antihypertensive activities.	[71]
3	Glycyl-l-valine	Analgesic, antipyretic, antioxidant, and anti-inflammatory activities.	[72]
4	Uridine	Neuroprotective activity, pyrimidine metabolism, antidepressant, and antiepileptic actions.	[73]
5	Tetradecanoic acid	Antipruritic, antifungal, anti-infective, and antioxidant activities.	[74]
6	Hexadecanoic acid	Antioxidant, antibacterial, anthelmintic, and antifungal activities.	[75]
7	Octadecanoic acid	Antifungal, antitumor, antibacterial, and antioxidant activities	[76]
8	Eicosanoic acid	Anti-abortifacient, antioxidant, antibacterial, analgesic, and antipyretic activities.	[77]
9	Docosanoic acid	Antipruritic, antioxidant, and anaesthetic activities	
10	Tetracosanoic acid	Antibacterial activity.	[78]
11	Hexadecanoic acid, methyl ester	Antispasmodial, antioxidant, and antiabortifacient activities.	[79]
12	Decanoic acid, 2-propenyl ester	Analgesic, antipyretic, antibacterial, antifungal, and anti-inflammatory activities.	[80]
13	*cis*-9-Octadecenoic acid	Antioxidant, anti-inflammatory, antitumor, antispasmodial, and antimicrobial activities.	[81]
14	9-Octadecynoic acid	Antifungal, cytotoxicity, antiasthmatic, and antidepressant activities.	[82]

**Table 5:** Compounds isolated from *Rhoicissus* species.

Plant Name	Class	Number	Name	Biological Activity	Reference
*R. digitata*	Triterpenoids	21	12,13-Dehydrolupeol	Anti-inflammatory, antitumor, chemopreventive, hepatoprotective, cardioprotective, and antiarthritic	[44,84]
		32	β-Sitosterol	Antidiabetic, neuroprotective, chemoprotective agent, antioxidant, anti-inflammatory, hypocholesterolemic, inducing apoptosis, angiogenic, anthelminthic, and immunomodulatory	[44,85]
		31	Oleanolic acid	Anticancer, antiosteoporosis, antiobesity, antidiabetic, lipid-lowering, anti-inflammatory, antioxidant, immune-regulatory, and hepatoprotective effects	[44,86]
	Flavonoids	34	(+)-Catechin	Antioxidant, antimicrobial, antimutagenic, anticarcinogenic, and cardioprotective	[44,87]
		22	Quercetin	Antioxidant (peroxonitrite (ONOO^−^) half maximal inhibitory concentration (IC_50_) = 8.6 µM; 2,2-diphenyl-1-picrylhydrazyl (DPPH) IC_50_ = 27.6 µM), anti-inflammation, antiviral, antiobesity, and antidepressant, as well as preventing cancer, diabetes, asthma, hypertension, and cardiovascular diseases	[44,88]
*R. tomentosa*	Terpenoids	23	3β-Taraxerol	Antidiabetic, anti-inflammatory	[44,89]
		24	Stigmasterol	Antiosteoarthritic, antihypercholestrolemic, cytotoxicity, antitumor, hypoglycaemic, antimutagenic, antioxidant, anti-inflammatory, and central nervous system (CNS) effects	[44,90]
		25	Oleanolic acid		[44]
		32	β-Sistosterol		[44]
	Flavonoids	25	Quercetrin		[44]
		34	(+)-Catechin		[44]
		26	Aromadendrin-7-*O*-β-glucopyranoside		[44]
	Alkaloid	27	Pheophytin	Neuroprotective, antimutagenic, anti-inflammatory	[44,91]
	Carotenoid	28	Lutein	Antioxidant, antiarthritis, anti-inflammatory, hepatoprotective, cardioprotective, anticataract, antidiabetic, anticancer, and bone remodelling activities	[44,92]

## Data Availability

Not applicable.

## Abbreviations

ANOVA	one-way analysis of variance
ALT	alanine amino transferase
ASP	aspartate amino transferase
ATP	adenoside triphosphate
BHA	butylated hydroxyanisole
BHT	butylated hydroxy toluene
CCl_4_	carbon tetrachloride
COX	cyclooxygenase
DMSO	dimethyl sulfoxide
DNA	deoxyribonucleic acid
G-6-Pase	glucose-6-phosphate
LOX	lipo-oxygenase
LPO	lipid peroxide
MIC	minimum inhibitory concentration
STI	sexually transmitted infection
TGF-β1	transforming growth factor-β1
UTI	urinary tract infection
